# Supplementary material for: Nanoparticle-Enabled Combination Therapy Showed Superior Activity against Multi-Drug Resistant Bacterial Pathogens in Comparison to Free Drugs
Source: Nanomaterials (Basel). 2022 Jun 24;12(13):2179. doi: 10.3390/nano12132179 (PMC9268018; doi:10.3390/nano12132179)
Supplement: Supplementary file 1 [file nanomaterials-12-02179-s001.zip › nanomaterials-1751508-supplementary.pdf]

nanomaterials

Supplementary Material

Nanoparticle-Enabled Combination Therapy Showed Superior Activity against Multi-Drug Resistant Bacterial Pathogens in Comparison to Free Drugs

Amarpreet Brar [1,2], Satwik Majumder [1,2], Maria Zardon Navarro [2,3], Marie-Odile Benoit-Biancamano [2,3], Jennifer Ronholm [1,4] and Saji George [1,2,*]

1 Department of Food Science and Agricultural Chemistry, Macdonald Campus, McGill University, 21111 Lakeshore, Ste Anne de Bellevue, QC H9X 3V9, Canada, amarpreet.brar@mail.mcgill.ca (A.B.); satwik.majumder@mail.mcgill.ca (S.M.); jennifer.ronholm@mcgill.ca (J.R.)
2 Swine and Poultry Infectious Diseases Research Center (CRIPA), Faculté de Médecine Vétérinaire, Université de Montréal, 3200 Sicotte, Saint-Hyacinthe, QC J2S 2M2, Canada, maria.zardon@umontreal.ca (M.Z.N.); marie-odile.benoit-biancamano@umontreal.ca (M.-O.B.-B.)
3 Research Group on Infectious Diseases in Production Animals (GREMIP), Department of Pathology and Microbiology, Faculté de médecine vétérinaire, , Université de Montréal, 3200 Sicotte, Saint-Hyacinthe, QC, Canada
4 Department of Animal Science, Macdonald Campus, McGill University, 21111 Lakeshore Ste Anne de Bellevue, QC H9X 3V9, Canada
* Correspondence: saji.george@mcgill.ca; Tel: +514-398-7920; Fax: +514-398-7990

Table S1. Antibiotics and their corresponding concentrations in the disks used in the determination of antimicrobial susceptibility of tested isolates.

Antibiotic	Disk Content
Apramycin (APR)	15 µg
Ampicillin (AMP)	10 µg
Amikacin (AK)	30 µg
Bacitracin (B)	10 iu
Cefotaxime (CTX)	30 µg
Cefazolin (CZ)	30 µg
Chloramphenicol (C)	30 µg
Ciprofloxacin (CIP)	5 µg
Colistin (CT)	10 µg
Erythromycin (E)	15 µg
Gentamycin (CN)	10 µg
Kanamycin (K)	30 µg
Lincomycin (MY)	2 µg
Neomycin (N)	30 µg
Ofloxacin (OFX)	5 µg
Penicillin (P)	10 iu
Streptomycin (SH)	100 µg
Spectinomycin (S)	10 µg
Tetracycline (TE)	30 µg
Ticarcillin (TIC)	75 µg
Tilmicosin (TIL)	15 µg
Tobramycin (TOB)	10 µg
Trimethoprim (SXT)	25 µg
Vancomycin (VA)	30 µg

The antimicrobial susceptibility of the isolates was determined using the Kirby-Bauer disk diffusion assay [1]. Isolates were cultured overnight at 37 °C in MHB. The cells were maintained at McFarland 0.5, and 10 µL of the inoculum was spread onto MH agar plates using a spreader. The disks containing the antibiotics were then placed on the lawn of bacteria using forceps and the plates were incubated for 18 h at 37 °C. The diameter of the zone of inhibition was measured, and the isolates were categorized as resistant or susceptible according to CLSI guidelines [2].

1. Resazurin Assay

Resazurin was used to measure bacterial viability [3]. Briefly, 30 µL of 0.5% resazurin solution was added to each well, incubated for 2 h at 37 °C, and read at 530/590 (excitation/emission) nm using a plate reader. *S. aureus* ATCC 25923 and *E. coli* ATCC 25922 were used as quality control strains to compare the MICs.

2. Determination of Efflux Activity

Efflux activity of the isolates was measured as described previously [4]. Briefly, bacterial cells cultured overnight at 37 °C, were washed thrice in phosphate buffered saline (PBS) (1X) and adjusted to McFarland 1.0. Ethidium bromide (EtBr) (3 µg/mL) was added to the bacterial suspension. Subsequently, EPIs - 3 µg/mL carbonyl cyanide m-chlorophenyl hydrazone (CCCP) for *Salmonella* and 30 µg/mL chlorpromazine (CPZ) for *Staphylococcus* isolates were added to allow maximum intracellular accumulation of EtBr under shaking at 25 °C for 60 min. Cells were then resuspended in 1 mL of PBS and transferred to a 96–well plate. Glucose (0.4% v/v) was added to trigger the efflux of EtBr and fluorescence was measured continuously for 40 min at 37 °C using a plate reader at 530–590 nm (excitation/emission) (SpectraMax i3, Molecular Devices, San Jose, CA, USA). Time ($T_{1/2}$) taken for the extrusion of half the amount of EtBr was measured. $T_{1/2}$ of isolates was compared based on which the efflux activity was determined. *S. aureus* 10812464, originally isolated from a case of bovine mastitis and known to have efflux activity as seen in our previous studies, and *S. enterica* ser. Typhimurium SL1344 was used as a positive control.

Amongst *Salmonella* isolates, *S. enterica* ser. Dublin showed the highest efflux activity (extrusion of EtBr), followed by *S. enterica* ser. Choleraesuis and *S. enterica* ser. Typhimurium (Figure S1(A)), with $T_{1/2}$ for EtBr extrusion as 241.56 s, 323.23 s, and 413.43 s respectively. Among *Staphylococcus* isolates, *S. aureus* M12 had the highest efflux activity, followed by *S. aureus* M17 and *S. hyicus* M43, with $T_{1/2}$ for EtBr extrusion as 240.4 s, 448.5 s, and 885.3 s, respectively. No efflux activity was observed for *S. hyicus* M48 (Figure S1B).

3. Beta-Lactamase Assay

Bacterial isolates grown overnight were adjusted to McFarland 1.0. Ampicillin (50 µg/mL) was added to the cell suspension and incubated at 37 °C for 3 h. Cells were then centrifuged at 9,000 × *g* for 10 min and washed in sodium phosphate buffer (pH 7.0). Pelleted cells were resuspended in buffer, sonicated on ice for 3 min, and centrifuged at 17,500 x *g* for 25 min. The cell-free extract was used for Nitrocefin assay [3]. Nitrocefin (abcam, Canada) solution of 10 µL was mixed with 10 µL of the cell-free extract, and the final volume was adjusted to 100 µL in wells of a 96-well plate. The absorbance was detected in kinetic mode for 10 min at 390 nm using a plate reader. The enzyme activity was calculated using the formula:

$$\text{beta-lactamase enzyme activity} = \frac{S_a}{\text{Reaction time X } S_v} \quad (1)$$

where S_a is the amount of nitrocefin (in µM) hydrolyzed in the unknown sample between t_1 and t_2 of the standard curve; reaction time is the time difference of t_1 and t_2, and S_v is the sample volume (in mL). Beta-lactamase activity is reported as U/mL.

All the tested isolates, except *S. hyicus* M48 showed beta-lactamase activity (Figure S1C). The highest beta-lactamase activity was observed in *S. enterica* ser. Typhimurium (84.73 U/mL), followed by *S. enterica* ser. Choleraesuis (72.62 U/mL) and by *S. enterica* ser. Dublin (67.52 U/mL). The least beta-lactamase activity was observed in *S. aureus* M17 (52.12 U/mL).

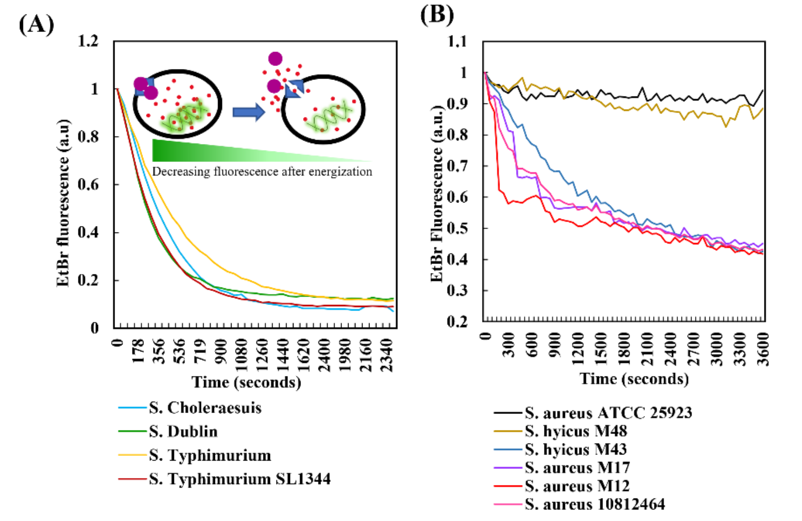

(C)

Strain	Nitrocefin hydrolysis (uM/min)ml-1
S. enterica ser. Typhimurium	84.73
S. enterica ser. Cholcracsuis	72.62
S. enterica ser. Dublin	67.52
S. aureus M12	67.1
S. hyicus M43	63.85
S. aureus M17	52.12
S. hyicus M48	No activity
S. aureus ATCC 25923	No activity
E. coli ATCC 25922	No activity

Figure S1. Efflux and beta-lactamase enzyme activity: Efflux pump activity in *Salmonella* (**A**) *and Staphylococcus* (**B**) isolates was assessed using EtBr as the substrate. Extrusion of EtBr was measured in kinetic mode by measuring the fluorescence at 530–590 nm (excitation/emission) after energizing the cells. (**C**) Tabular data showing beta-lactamase enzyme activity in *Staphylococcus* and *Salmonella* isolates.

4. Biofilm Formation

The ability of the bacterial isolates to form a biofilm was determined using a crystal violet assay [5]. Briefly, bacterial cells were grown overnight at 37 °C under shaking. The cell suspensions (0.5 McFarland) of 200 µL were added to the wells of two 96-well plates. One plate was incubated for 24 h whereas, the other for 48 h. The wells were washed with saline 3× to remove any non-adherent cells and the adherent cells were fixed with 200 µL of 99% methanol. After 15 min, the methanol was removed, and the plates were air-dried. A 200 µL aliquot of crystal violet dye (0.2%) was added to the wells. After 40 min, the wells were washed, resuspended with 200 µL of acetic acid (33%), and absorbance was measured at 570 nm. The ability of bacterial isolates to form a biofilm was categorized as strong, moderate, weak, or no biofilm formation based on critical optical density (OD_c) compared to the optical density of the sample (OD_s). OD_c was calculated using the arithmetic mean of the absorbance of negative control and three times the addition of standard deviation. The biofilm-forming ability was categorized as no biofilm formation ($OD_s < OD_c$), weak biofilm formation ($OD_c < OD_s \leq 2OD_c$), moderate biofilm formation ($2OD_c < OD_s \leq 4OD_c$), and strong biofilm formation ($4OD_c < OD_s$).

The biofilm formation of bacterial isolates is compared in Figure S2. After 24 h, *S. enterica* ser. Choleraesuis, *S. enterica* ser. Dublin and *S. enterica* ser. Typhimurium SL1344 formed no biofilm while weak biofilm formation was observed for *S. aureus* M17, *S. hyicus* M48, and *S. enterica* ser. Typhimurium. *S. aureus* M12 and *S. hyicus* M43 formed moderate biofilm. After 48 h of incubation, *S. aureus* M12 and *S. hyicus* M43 formed stronger biofilm while there was no change in biofilm-forming ability for *S. hyicus* M48 and *S. enterica* ser. Typhimurium even after 48 h. *S. aureus* M17 formed weak biofilm, while no biofilm formation was observed in *S. enterica* ser. Choleraesuis and *S. enterica* ser. Dublin even after 48 h.

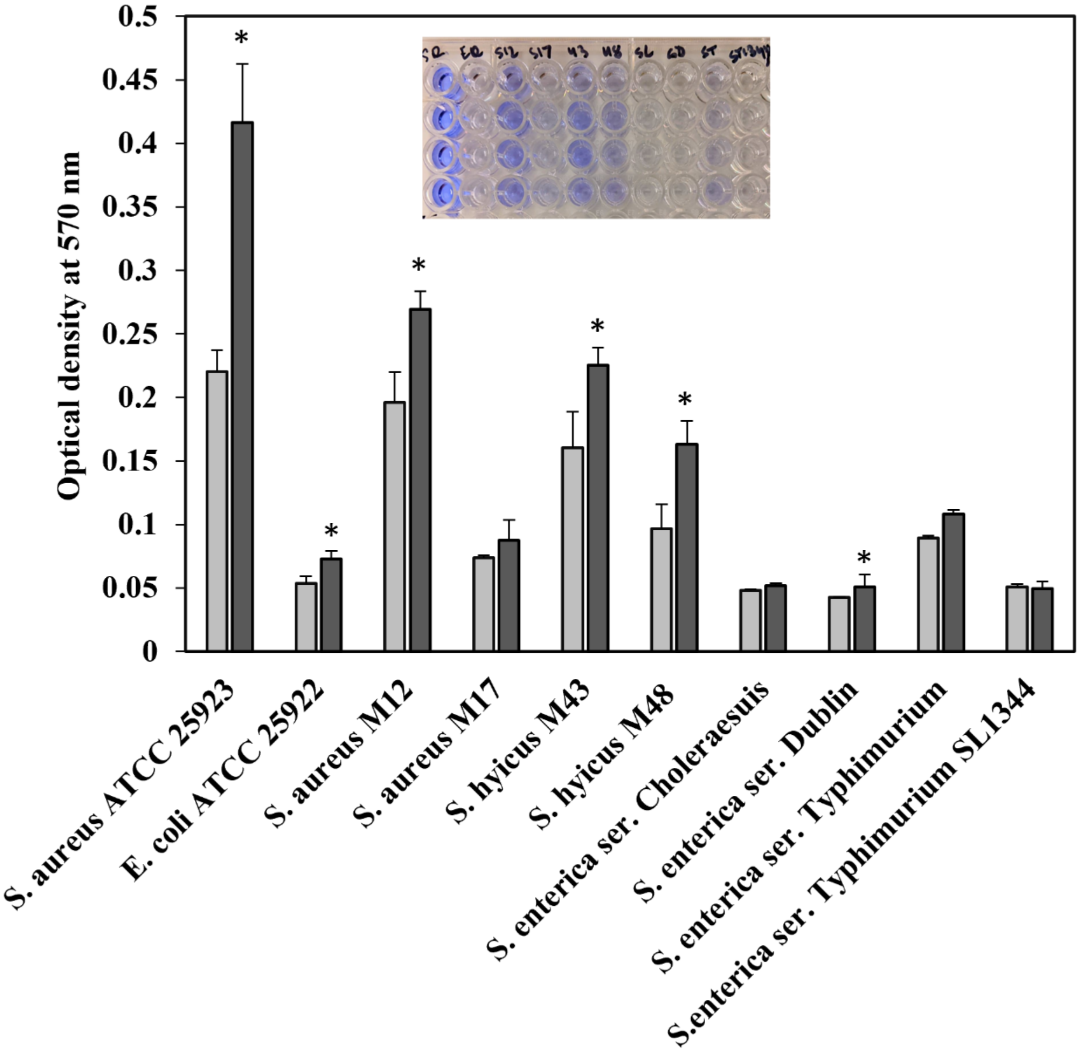

Figure S2. Biofilm formation assay: Biofilm formation of *Staphylococcus* and *Salmonella* isolates from animal farms measured using crystal violet assay after 24 h (light grey) and 48 h (dark grey). Inlet shows the image of one of the plates used for biofilm assay. A significant difference in biofilm formation ability ($p < 0.05$) is marked as '*'.

5. Hemolysis of Blood Agar Plates

Bacterial isolates were tested for the manifestation of hemolysins as detailed previously [6]. Briefly, bacterial isolates were grown overnight in MHB and streaked onto blood agar plates (TSA with 5% defibrinated sheep blood). The plates were incubated at 37 °C for 24 h. The pattern of hemolysis was detected by visual inspection for the translucency around the bacterial colony.

Results of hemolysis are summarized in tabular data Figure S3A**.** A clear zone of hemolysis (beta-hemolysis) was observed in S. *aureus* M12 (Figure S3B), *S. aureus* M17, S. *hyicu*s M43 while partial hemolysis or green discoloration (α-hemolysis) was observed in S. *hyicus* M48. *Salmonella* isolates showed green discoloration (Figure S3B) around the bacterial colonies, and thus categorized as hemolysin-producing isolates.

(A)

	α hemolysis	β hemolysis
S. aureus ATCC 25923	-	+
S. *aureus* M12	-	+
S. aureus M17	-	+
S. hyicus M43	-	+
S. hyicus M48	+	-
	**Hemolysis**	**No hemolysis**
S. enterica ser. Choleraesuis	+	-
S. enterica ser. Dublin	+	-
S. enterica ser. Typhimurium	+	-

(B)

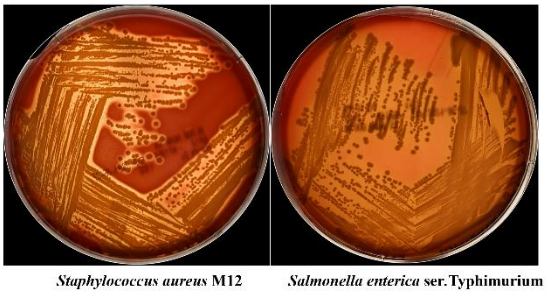

Figure S3. Hemolysis assay: Bacterial isolates were streaked onto tryptic soy agar plates containing 5% defibrinated sheep blood and incubated overnight. Hemolysis activity was assessed based on characteristic translucency around bacterial colonies. (**A**) Tabular data summarizing the type of hemolysis activity in tested isolates. (**B**) *S. aureus* M12 produced translucent halo around the cultured colonies – beta-hemolysis, *S. enterica* ser. Typhimurium showed hemolysis (green discoloration).

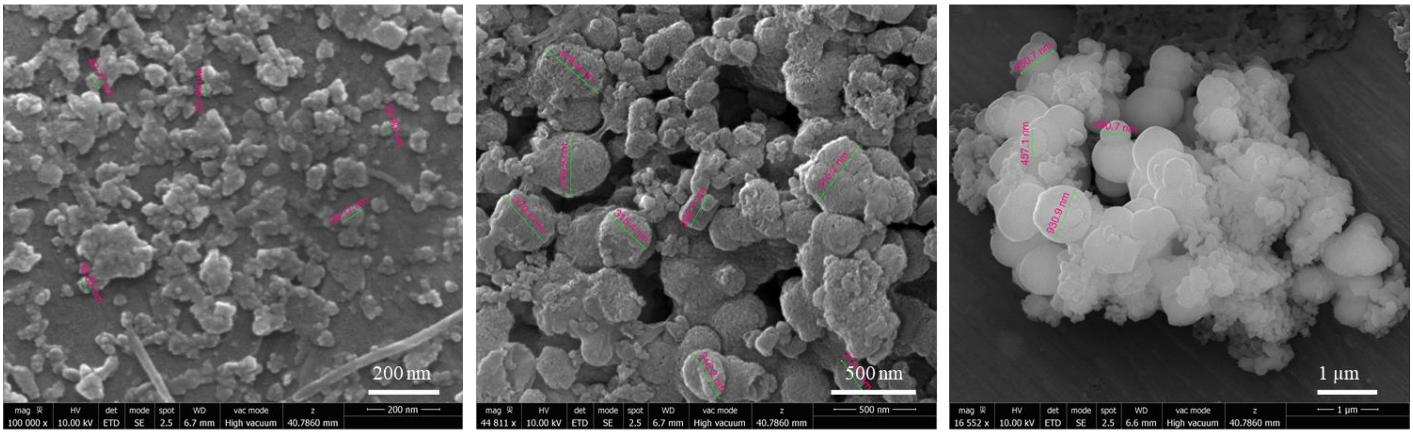

Figure S4. SEM images of Ch–TET NPs, Si–CPZ NPs, and CMD with marked dimensions.

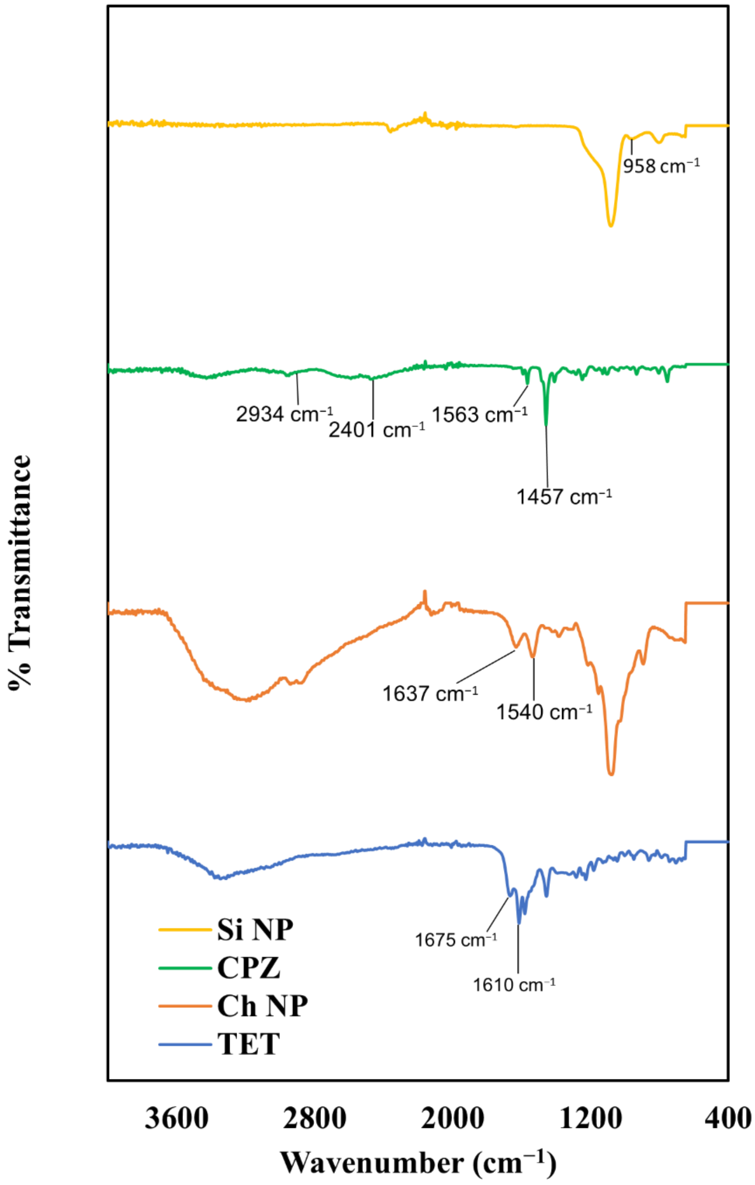

Figure S5. FTIR spectra of Si NP, CPZ, Ch NP, and TET.

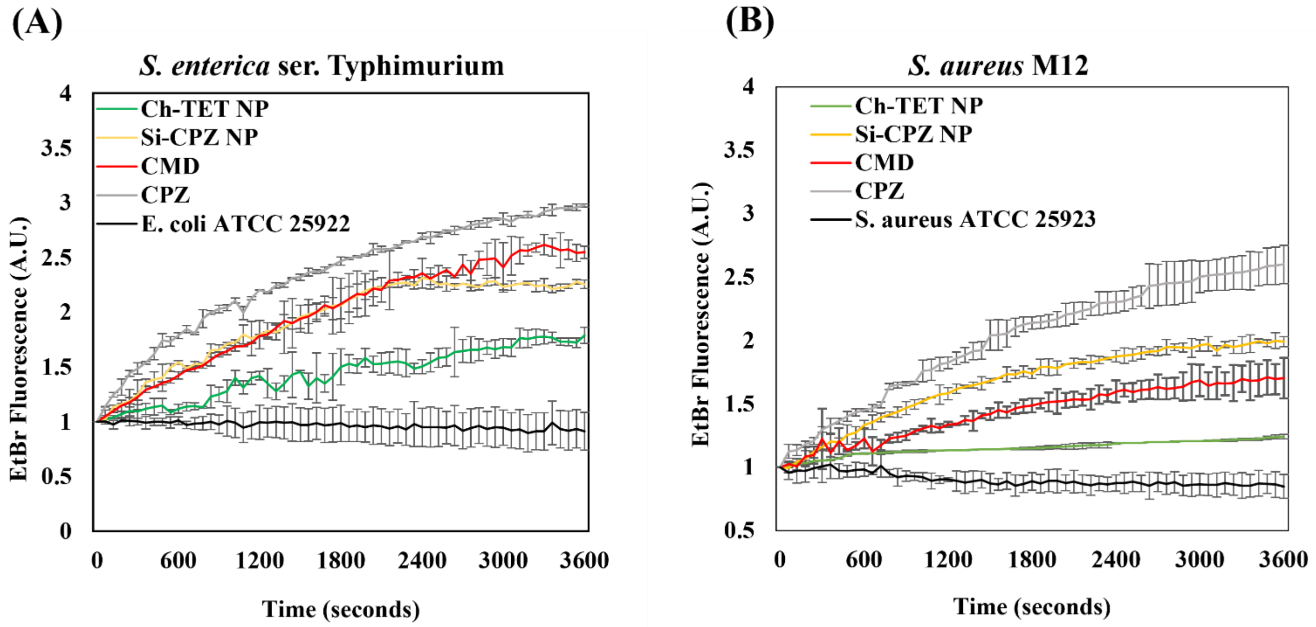

Figure S6. EtBr accumulation in bacterial cells**.** Accumulation of EtBr in *S. enterica* ser. Typhimurium (**A**) and *S. aureus* M12 (**B**) treated with synthesized nanoparticles determined by measuring fluorescence (530/590) nm.

6. EtBr Accumulation Assay

It was observed that in *S. enterica* ser. Typhimurium, EtBr accumulation was fastest when treated with Si–CPZ NP with $t_{\frac{1}{2}}$ of 1398 s. The CMD also led to fast accumulation of EtBr with $t_{\frac{1}{2}}$ of 1771 s. Ch–TET NP treated cells showed very slow accumulation with $t_{\frac{1}{2}}$ of >3600 s. *S. enterica* ser. Typhimurium treated with CPZ was used as a positive control. In case of *S. aureus* M12, Si–CPZ NP treated bacterial cells showed fastest accumulation of EtBr ($t_{\frac{1}{2}}$ of 1793 s). CMD also successfully led to accumulation of EtBr ($t_{\frac{1}{2}}$ of 2176 s). Extremely slow accumulation of EtBr was observed for bacterial cells treated with Ch–TET NP ($t_{\frac{1}{2}}$ >3600 s). *S. aureus* M12 treated with CPZ was used as positive control.

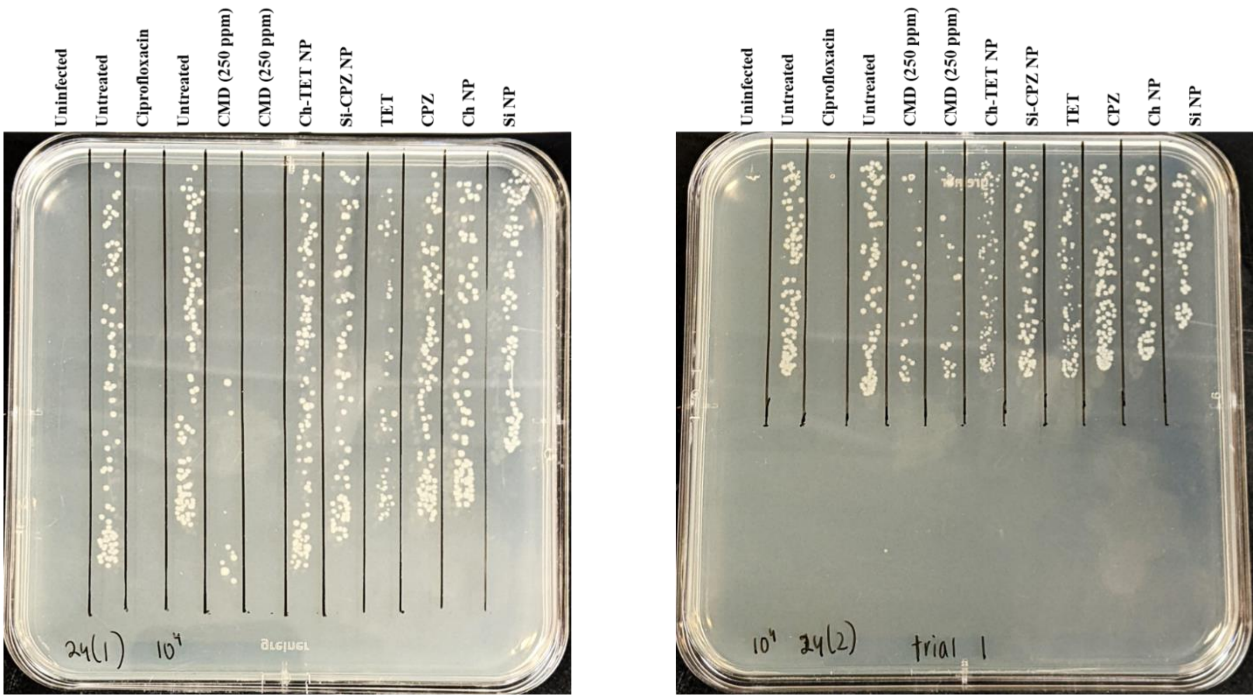

Figure S7. Determination of CFU/mL. Image of plates used to determine CFU/mL of lysates of infected Caco–2 cells treated with nanoparticle sample

References

1. Hudzicki, J. *Kirby-Bauer Disk Diffusion Susceptibility Test Protocol*. American Society for Microbiology 2009. https://asm.org/getattachment/2594ce26-bd44-47f6-8287-0657aa9185ad/Kirby-Bauer-Disk-DiffusionSusceptibility-Test-Protocol-pdf.pdf (accessed on 14 March 2021).
2. Clinical Laboratory Standards Institute. *Performance Standards for Antimicrobial Susceptibility Testing*; 2021. https://clsi.org/standards/products/microbiology/documents/m02/ (Accessed on 24 October 2020).
3. Majumder, S.; Jung, D.; Ronholm, J.; George, S. Prevalence and mechanisms of antibiotic resistance in Escherichia coli isolated from mastitic dairy cattle in Canada. *BMC Microbiol.* **2021**, *21*, 222.
4. Paixão, L.; Rodrigues, L.; Couto, I.; Martins, M.; Fernandes, P.; de Carvalho, C.C.C.R.; Monteiro, G.A.; Sansonetty, F.; Amaral, L.; Viveiros, M. Fluorometric determination of ethidium bromide efflux kinetics in Escherichia coli. *J. Biol. Eng.* **2009**, *3*, 18. https://doi.org/10.1186/1754-1611-3-18.
5. Xu, Z.; Liang, Y.; Lin, S.; Chen, D.; Li, B.; Li, L.; Deng, Y. Crystal violet and XTT assays on Staphylococcus aureus biofilm quantification. *Current microbiology* **2016**, *73*, 474-482.
6. Buxton, R. Blood agar plates and hemolysis protocols. *American Society for Microbiology* **2005**, 1-9. https://asm.org/Protocols/Blood-Agar-Plates-and-Hemolysis-Protocols#:~:text=Blood%20Agar%20Plates%20and%20Hemolysis%20Protocols,-Download%20the%20Protocol&text=Many%20species%20of%20bacteria%20produce,based%20on%20their%20hemolytic%20properties. (Accessed on 20 February 2021).
